# Modifying enzyme replacement therapy – A perspective

**DOI:** 10.1111/jcmm.17653

**Published:** 2022-12-25

**Authors:** Philipp Schaible

**Affiliations:** ^1^ Faculty of Biology University of Freiburg Freiburg Germany

**Keywords:** B cell, Cell engineering, T cell, Protein replacement

## Abstract

Several diseases are caused by the lack of functional proteins, including lysosomal storage diseases or haemophilia A and B. Patients suffering from one of these diseases are treated via enzyme replacement therapies to restore the missing protein. Although this treatment strategy prevents some disease symptoms, enzyme replacement therapies are very expensive and require very frequent infusions, which can cause infusion adverse reactions and massively impair the quality of life of the patients. This review proposes a technology to sustainably produce proteins within the patient to potentially make frequent protein‐infusions redundant. This technology is based on blood circulating immune cells as producers of the needed therapeutic protein. To ensure a stable protein concentration over time the cells are equipped with a system, which induces cell proliferation when low therapeutic protein levels are detected and a system inhibiting cell proliferation when high therapeutic protein levels are detected.

## 1 INTRODUCTION

Lysosomal storage diseases are caused by loss‐of‐function mutations in enzymes, which normally degrade proteins (e.g., glycosaminoglycan) to prevent pathological accumulations.^1^ These accumulations can cause bone deformities, anaemia and seizures.^2^ Enzyme replacement therapy is used to treat patients with lysosomal storage disease by constantly supplying functional enzymes.^3^ Therefore, recombinant enzymes are produced (in human fibroblasts, Chinese hamster ovary cells or plant cells^4^), purified and intravenously injected into the patient. The first lysosomal storage disease treated effectively with enzyme replacement therapy was Gaucher's disease,^5^ followed by various others.^3^

Besides lysosomal storage diseases, enzyme replacement therapies are successfully used to treat haemophilia A and B.^6^ Haemophilia A and B are bleeding disorders, caused by a lack or reduction of coagulation factor VIII (haemophilia A) or IX (haemophilia B).^7^ Haemophilia A and B can cause hemarthosis, hematuria and intracerebral haemorrhages. In patients with very low factor VIII or IX concentrations, symptoms can occur independently from a triggering trauma; in patients with higher concentrations, the symptoms are milder and need to be triggered by moderate traumas.^7^ Early treatment strategies were based on an on‐demand therapy of recombinant factor VIII concentrates,^8^, ^9^ which however was unable to prevent arthropathy and muscle atrophy in patients.^10^ Hence, the treatment strategy was pivoted to supply factor VIII/IX regularly.^11^

Despite their success, the current enzyme replacement therapies have some disadvantages, like the need of frequent infusions caused by the limited half‐life time of the enzymes. Haemophilia patients have to infuse factor VIII/IX every 2 or 3 days. Patients with mucopolysaccharidoses (a group of lysosomal storage diseases) need weekly infusions, which take around 3–4 h every time. Additionally, these infusions can elicit infusion adverse reactions, ranging from rash, angioedema and bronchoconstriction to anaphylaxis.^12^, ^13^, ^14^ Although the majority of infusion adverse reactions are mild, most infusions are conducted in a hospital, which puts an additional burden on patients. Furthermore, enzyme replacement therapies are extremely expensive, with annual costs of up to several hundred thousand dollars.^15^

## 2 PROPOSED TECHNOLOGY

The treatment of the above‐mentioned diseases might be improved by a system, which enables the durable expression of a therapeutically needed protein at a constant concentration. Therefore, this article proposes a self‐regulating system based on circulating blood cells (B or T cells) to produce a therapeutically needed protein. To ensure a stable protein expression over time, these carrier cells need a self‐regulating system to counteract cell loss and keep cell and protein concentration stable.

Each carrier cell might be engineered to harbour the following components:

1. A therapeutic protein is needed, which is constantly produced and secreted into the bloodstream.
2. To counteract the fade away of carrier cells over time accompanied by the decrease of therapeutic protein concentration, a system is needed, increases the cell number to compensate cell loss and low therapeutic protein concentration. Therefore, a so‐called helper protein might be expressed and secreted into the bloodstream, able to bind to a helper protein receptor. Thereby the helper protein receptor might be activated to subsequently promote cell proliferation.
3. To prevent a self‐amplifying system – in which higher carrier cell numbers cause higher helper protein concentration, ultimately causing higher cell numbers – a system is needed to inhibit cell proliferation when high therapeutic protein concentration is detected. This system might include a receptor able to sense the amount of available therapeutic protein in the bloodstream. Activation of this therapeutic protein receptor might induce the expression of a short hairpin RNA (shRNA), which elicits RNA interference to inhibit the expression of the helper protein. Hence, high cell numbers (causing high therapeutic protein concentration and therapeutic protein receptor activation) silence the expression of the helper protein expression and its proliferative effects.

**FIGURE 1:**
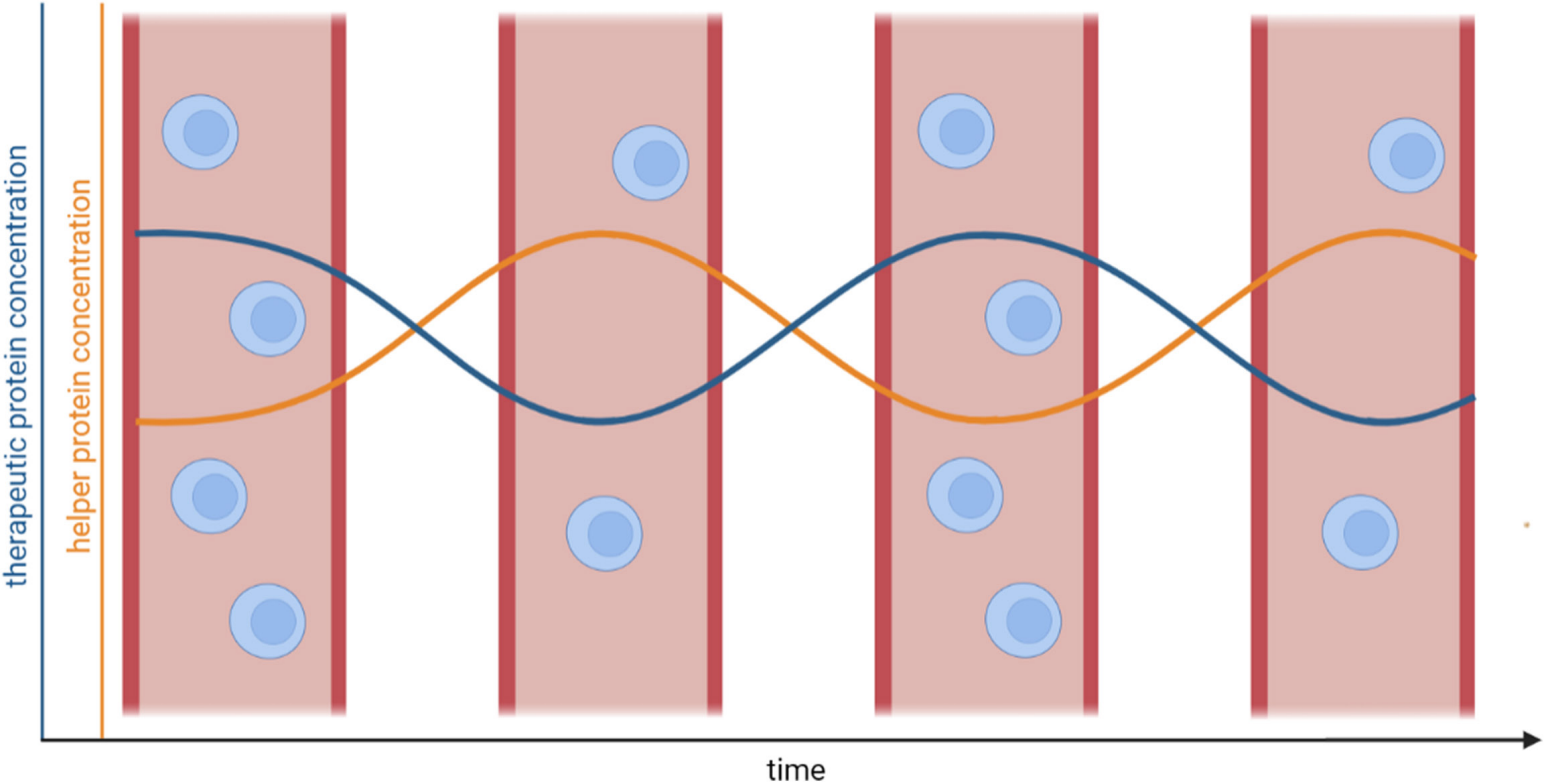
Temporal development of carrier cell‐, therapeutic protein‐ and helper protein‐concentration. A gradual decrease in carrier cell number causes lower therapeutic protein concentration. Decreased protein concentration then causes increased helper protein concentration and consequently increases the cell number.

**FIGURE 2:**
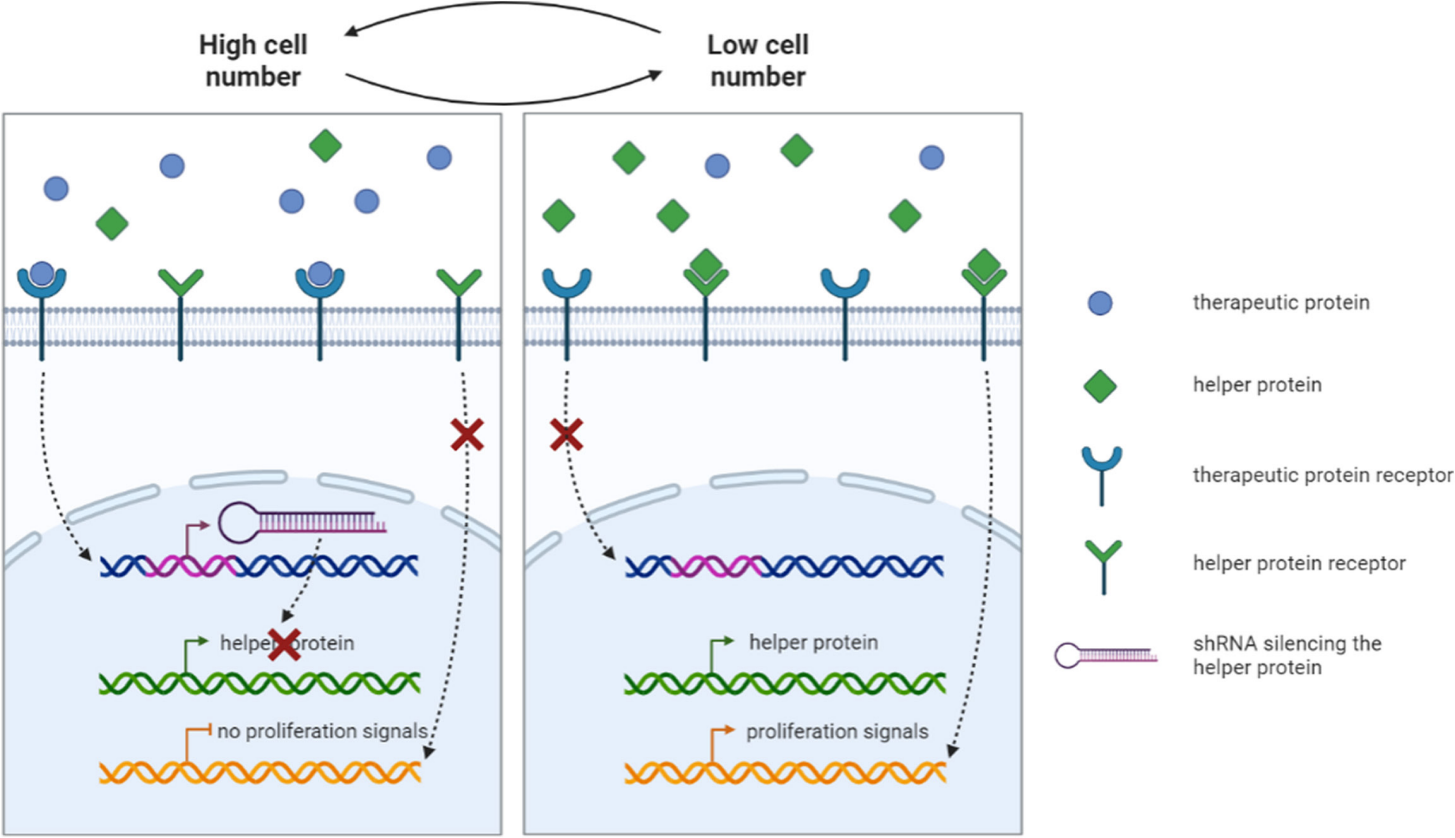
Overview of the technology. High cell numbers and concomitant high therapeutic protein concentration activate the therapeutic protein receptor. This activation causes the production of a short hairpin RNA (shRNA), which then silences the expression of the helper protein. The absence of a helper protein prevents the activation of the helper protein receptor and the cells do not proliferate. Low cell numbers and concomitant low therapeutic protein concentration do not activate the therapeutic protein receptor, hence no shRNA is produced. The lack of shRNA causes the production of helper protein, which then activates the helper protein receptor, causing cell proliferation.

Thereby, a self‐regulating system might be created in which low carrier cell numbers (low therapeutic protein concentration) cause the expression of a helper protein, which then increases the cell number. In contrast, high cell numbers (high therapeutic protein concentration) cause the inhibition of the helper protein and its proliferative effects (see Figures 1 and 2).

## 3 COMPONENTS OF THE SELF‐REGULATING SYSTEM IN DETAIL

### 3.1 Carrier cell

Ideally, the carrier cells must be (i) accessible for genetic engineering, (ii) long‐living, (iii) able to durable and robustly express the integrated genes, (iv) circulates within the blood system and (v) scalable industrial manufacturing must be possible.

B or T cells might be potential carriers for the here proposed technology. B cells, especially plasma cells, have the advantage that they are able to produce and secrete high amounts of proteins (antibodies),^16^ which might enable them to produce high amounts of the therapeutic protein.^17^, ^18^ Furthermore, B cells are able to clonally expand and differentiate into long‐lived plasma and memory cells. However, challenges in in vitro culturing, cell expansion and the low engineering efficiency in primary B cells might hamper their use^19^ and favour the use of T cells.

As B cells, T cells can clonally expand after stimulation^20^ and are able to produce high amounts of proteins (especially effector T cells).^21^ Furthermore, T cells are easily accessible and its culturing, engineering and infusion into patients following good manufacturing processes is well established, due to the longstanding experience with chimeric antigen receptor (CAR)‐T cells.^22^, ^23^ Additionally, T cells are able to resist oncogenic transformation.^24^ Hence, this review will focus on T cells and analyse in more depth which phenotypes might be used.

Noteworthy, B and T cells and their different phenotypes might have different efficiency in producing and secreting the therapeutic protein. To compensate these differences, the system is designed to adjust to a specific therapeutic protein concentration rather than to a specific carrier cell number. In case of higher production and secretion efficiency, lower cell numbers are needed to reach the targeted therapeutic protein concentration. In case of lower efficiency, higher cell numbers are needed.

T cells can be differentiated into several phenotypes including naïve, effector, central memory and stem cell memory T cells.^25^ For the here proposed technology, a phenotype able to proliferate (to compensate cell loss) and persist a long time in vivo is needed. Memory T cells might be promising since they have stem cell‐like features, including telomerase expression^26^, ^27^ and a long half‐life time (between 1 and 12 months^28^, ^29^). Additionally, it was shown that specific memory T cell clones can survive much longer than 12 months since T cell populations specific for particular viruses are present even decades after the patient was vaccinated.^29^ A constant homeostatic turnover within the population of circulating memory T cells is thought to self‐renew the cells and sustain the populations.^26^, ^27^ After antigen stimulation, memory T cells are able to massively proliferate and differentiate into effector T cells.^30^

Since a memory phenotype might be beneficial for the here proposed technology, the infused T cells might be supported to maintain a memory‐like phenotype and to transit back into it after proliferation. IL‐7 and IL‐15 are essential interleukins for the survival and self‐renewal of memory T cells.^31^ It is speculated that IL‐7 mainly supports the survival of the memory T cells and that IL‐15 supports self‐renewal and homeostatic turnover.^32^, ^33^ Furthermore, both interleukins participate in the transition from effector T cells into memory T cells.^32^, ^33^ It was shown that increasing the IL‐7 or IL‐15 level caused higher memory T cell numbers and longer memory T cell survival.^34^, ^35^ In contrast, low IL‐7 and IL‐15 levels decreased memory T cell numbers.^36^, ^37^ Hence, to support a memory phenotype of the infused cells, IL‐7 and IL‐15 pathways might be upregulated by overexpressing the IL‐7 and IL‐15 receptors. In line with the abovementioned effects of IL‐7 and IL‐15, the IL‐7 receptor (IL‐7Rα) is needed for memory T cell longevity^38^, ^39^ and effector T cells expressing high levels of the IL‐7Rα are more likely to survive after stimulation and to (re)build the population of memory T cells.^27^, ^38^, ^40^, ^41^ Additionally, mice lacking the IL‐15 receptor (IL‐15Rα) show reduced memory T cell numbers.^36^ Therefore, infused cells overexpressing the IL‐7Rα and IL‐15Rα might be more likely to express a memory‐like phenotype and to survive longer. Further potential genes to support longevity and a memory phenotype might be OX‐40,^42^ CD27^43^ and 4‐1BB.^44^

Furthermore, the engineered T cells might show an impairment of their inherent immune function, caused by the complex engineering and the burden of producing and secreting the various components of the proposed technology. However, the engineered T cells might still be activated by pathogens, resulting in cell proliferation. This increase in T cell number might cause overshooting therapeutic protein production and potentially cause toxicities. Hence, the immune function of the engineered T cells might be abolished by knocking out the T cell receptor (TCR), as successfully performed in various studies with allogeneic CAR‐T cells.^45^, ^46^, ^47^

### 3.2 Therapeutic protein

The used therapeutic protein is disease‐specific, must however be recognizable by its therapeutic protein receptor. As described earlier, diseases currently treated via enzyme replacement therapy might be a good fit for the here proposed technology. Hence, the above‐mentioned factor VIII (haemophilia A), factor IX (haemophilia B) or glucocerebrosidase (Gaucher's disease) might be used as a therapeutic protein. Further diseases currently treated via classical enzyme replacement therapies and potential candidates for the here proposed technology might be mucopolysaccharidosis (MPS) I, MPS II, MPS IV A, MPS VI, Fabry disease or Pompe disease.^3^, ^48^

### 3.3 Therapeutic protein receptor

The therapeutic protein receptor must be able to sense extracellular therapeutic proteins and subsequently activate the expression of an shRNA, able to silence the expression of the helper protein. Therefore a so‐called synNOTCH receptor might be used, initially designed by Lim et al. to precise the anti‐tumour activity of CAR‐T cells.^49^, ^50^, ^51^ This receptor is composed of an extracellular antigen‐specific single‐chain variable fragment, a Notch core region and an intracellular transcriptional factor. Antigen binding triggers several conformational changes, leading to the release of the intracellular transcription factor and ultimately causing transgene (shRNA) expression.^49^

### 3.4 Helper protein

To stimulate cell proliferation, the helper protein must be able to bind and activate the helper protein receptor without eliciting effects in cells, which do not belong to the here proposed system. Furthermore, the helper protein should be exclusively expressed by the infused cells to prevent unwanted proliferative signals and must not be immunogenic.

Hence, small peptides might be used as helper proteins, since they can be designed to exclusively bind to the helper protein receptor and are generally considered non‐immunogenic.^52^, ^53^ Furthermore, the engineered cells are then the exclusive source of the peptide. A further advantage is a long‐standing experience with bioinformatical and biomolecular technologies to design and test small peptides.^54^, ^55^

### 3.5 Helper protein receptor

The helper protein receptor must be able to bind the helper protein and subsequently induce the proliferation of the engineered cells. Thereby, low cell numbers (indicated by an increase of helper proteins in the bloodstream) would cause cell proliferation. CAR might be used as helper protein receptors.

The CARs contain an extracellular antigen‐binding domain composed of a single‐chain variable fragment, which might be engineered to bind the helper protein. Furthermore, the CARs have an intracellular signalling domain, which differs between the first, second and third CAR‐T cell generation. The intracellular signalling domain of the first CAR‐T cell generation contains the CD3ζ chain of the T cell co‐receptor. However, activation of the first CAR‐T cell receptor generation was unable to elicit a strong cytokine response and proliferation.^56^, ^57^ To improve CAR‐T cell activity of the second and third generation, costimulatory domains (CD28 or 4‐1BB in the second generation and both domains simultaneously in the third generation) were added to the intracellular signalling domain.^58^ Although the first generation of CAR‐T cell receptors was insufficient to trigger proliferation and cytokine expression strong enough to fight cancer, this mild activation might be a good fit to modestly stimulate the cells used in the here proposed technology. Alternatively, second‐generation CARs (with the 4‐1BB costimulatory domain) might be used, since they also show reduced effector functions compared to CAR‐T cells with CD28 as a costimulatory domain.^59^, ^60^

### 3.6 shRNA

The shRNA must be designed to silence the helper protein to suppress its proliferative effects during a condition of high cell numbers and high therapeutic protein concentration. shRNAs elicit the so‐called RNA interference to sequence‐specifically suppress the mRNA of a targeted protein.^61^ Therefore, shRNAs produce double‐stranded 19–25 nucleotide long small interference RNAs (siRNAs). These siRNAs are able to associate with the protein argonaute‐2 in the cytoplasm, which is part of the RNA‐induced silencing complex (RISC). Subsequently, one strand of the siRNA (passenger strand) is removed and the other strand (guide strand) navigates RISC to the mRNA target to degrade it.

Instead of suppressing the helper protein, the shRNA might be designed to target the helper protein receptor or two shRNAs might be used to target the helper protein and its receptor simultaneously.

## 4 PRODUCTION OF THE CARRIER CELLS

In case of T cells as carriers of the here proposed technology, the production process can be adopted from CAR‐T cells (reviewed by e.g., Vormittag et al.,^62^ and Abou‐El‐Enein et al.^63^). In brief, white blood cells are collected via leukapheresis, followed by cell washing to remove remaining platelets and red blood cells. Thereafter, T cells are activated by magnetic beads coated with CD3 and CD28.^64^ Alternatively, anti‐CD3 monoclonal antibodies are used in combination with IL‐2 (or a combination of IL‐7 and IL‐15, which ameliorates the generation of memory T cells).^65^ Then, genes are delivered into the cells via non‐viral or viral delivery systems, with viral transduction used in all commercially available CAR‐T cell therapies.^66^, ^67^, ^68^, ^69^, ^70^ The most common viral vectors are lenti‐ or retro‐viruses, which have a high transduction efficiency.^71^ Since these viruses integrate transduced genes into the host cells genome, their use causes the risk of oncogenic transformation.^72^ However, in the clinical use of CAR‐T cells oncogenic transformation was not reported to date.^62^ After gene delivery, CAR‐T cells are expanded, cryopreserved and transported to the patient, where the CAR‐T cells are thawed and infused.

## 5 CHALLENGES

### 5.1 Adjusting towards a specific therapeutic protein concentration

Each potentially treated disease needs a specific blood concentration of the particular therapeutic protein to reach the therapeutic window. Hence, the production of the therapeutic protein might be fine‐tuned by adjusting the affinity of the therapeutic protein receptor towards its ligand. If the receptor is adjusted towards high affinity, cell rejuvenation/proliferation is suppressed until a very low therapeutic protein concentration is reached. In contrast, if the receptor is adjusted towards low affinity, cell rejuvenation/proliferation is allowed until a high therapeutic protein concentration is reached. Alternatively, the expression of the therapeutic protein receptor might be modulated.

### 5.2 Preventing adverse effects

Safety mechanisms like off‐switches might be integrated to enable cell‐killing in case of toxicities or tumour formation. Off‐switches are fusion proteins used in CAR‐T cells, which contain, for example, iCasp9 (a suicide gene) and FK506‐binding protein (a protein binding the small molecule AP1903). After infusing AP1903 into the patient, iCasp9 is dimerized and activated, causing the apoptosis of CAR‐T cells. Thereby, more than 90% of the engineered T cells can be killed within 30 min.^73^ Alternatively to off‐switches, the T cells might be engineered to express so‐called elimination markers, normally not present on the surface of T cells. The engineered T cells can then be abolished via antibody‐dependent cytotoxicity.^74^ Potential elimination markers might be CD20 with rituximab as targeting antibody^75^ or EGFR with cetuximab.^76^

Besides toxicities caused by the engineered T cells, the patient might independently develop T cell lymphoma or other types of cancer, necessitating harsh treatment.^77^ This treatment might then impair or abolish the here proposed system. In this case, the patient must be treated via conventional enzyme replacement therapy during the period of the cancer treatment.

### 5.3 Complexity

The here proposed technology is characterized by high complexity, with at least five different transgenes needed (therapeutic protein, helper protein, shRNA, therapeutic protein receptor and helper protein receptor). This complexity might impair T cell production. However, T cell engineering is well established and the production of very complex CAR‐T cells is possible. Several different genes can be simultaneously integrated into allogenic CAR‐T cells (including the CAR, HLA‐E, safety‐switch and genes involved in arming the CAR‐T cell) and knocked‐out (including endogenous TCR, HLA class I, HLA class II and the programmed cell death protein 1).^78^

The most critical structures of the proposed technology are the therapeutic protein receptor and the shRNA. Losing one of these structures would create cells with a self‐amplifying system, driving cell proliferation. A loss of one of the other structures is less critical since it would either create cells unable to proliferate (in case of helper protein receptor loss); or would be compensated by other cells (in case of a therapeutic protein or helper protein loss). Cells unable to proliferate would progressively fade away and be replaced by functional cells.

### 5.4 Immunogenicity

Another potential problem might be, that the therapeutic protein, helper protein, therapeutic protein receptor and helper protein receptor might be immunogenic. Immunogenicity is a common feature of enzyme replacement therapies, causing the production of anti‐drug antibodies. A 90% of mucopolysaccharidoses I patients treated with Laronidase produce anti‐drug antibodies,^12^ 50% of mucopolysaccharidoses II patients treated with Idursulphase^79^ and almost all mucopolysaccharidoses II patients treated with either Elosulphase^80^ or Galsulphase.^81^ Same adverse reactions occur in haemophilia A patients treated with factor VIII concentrates, including the treatment with Kogenate (29.7%), Recombinate (31.0%) and ReFacto (33.0%).^82^ These frequently occurring anti‐drug antibodies might reduce the efficacy not only in enzyme replacement therapies,^83^, ^84^ but also in the here proposed technology.

Furthermore, patient's T cells might be activated by the therapeutic or helper protein, expressed on the surface of the infused cells. To prevent this adverse T cell activation, the infused cells might overexpress immunosuppressive factors like the ligand of PD‐1 (PDL‐1), which is an immunosuppressive receptor expressed on activated T cells to prevent massive immune activation and autoimmunity.^85^

### 5.5 Organ specificity

As in classical enzyme replacement therapy, the here proposed technology might secrete the therapeutic protein mainly into the circulatory system (especially applicable for the production of factor VIII or factor IX). Hence, it might be beneficial to engineer the T cells to remain within the circulatory system.

To extravasate the circulatory system, immune cells migrate through a barrier of endothelial cells and the endothelial cell basement membrane. This multi‐step process is divided into capture, rolling, activation, arrest, adhesion strengthening and paracellular or transcellular migration.^86^ The extravasation of T cells relies on the binding of T cell integrins (including the β1‐integrin VLA‐4 and the β2‐integrin LFA‐1) to adhesion molecules expressed by endothelial cells, including ICAM‐1, ICAM‐2 and VCAM‐1.^86^ Furthermore, it was shown that L‐selectin participates in T cell extravasation by binding its endothelial ligands CD34, GlyCAM‐1 and MadCAM‐1^87^, ^88^ and that L‐selectin‐deficiency impairs T cell extravasation.^89^ Additionally, mice with an LFA‐1 deficiency show impaired immune cell infiltration in the lymph nodes^90^ and the simultaneous blockade of LFA‐1 and VLA‐4 completely inhibits immune cell extravasation.^91^ Moreover, monoclonal antibodies are (natalizumab) and were (efalizumab, withdrawn 2009) used to suppress immune cell extravasation in inflammatory diseases like multiple sclerosis and Crohn's disease. Mechanistically, natalizumab suppresses the extravasation by binding VLA‐4^92^ and efalizumab by binding LFA‐1.^93^

Hence, a strategy to support therapeutic protein expression within the circulatory system might be to reduce T cell extravasation by knocking out the abovementioned adhesion molecules. However, certain diseases elicit organ‐specific symptoms (e.g., Fabry disease or Pompe disease)^94^ and might profit from the extravasation of the engineered cells into the affected tissue.

## 6 AGE‐RELATED DISEASES

Besides being an alternative treatment for diseases currently treated via enzyme replacement therapy, the proposed technology might be used more broadly to, for example, ameliorate age‐associated diseases.

Several blood‐circulating factors are dysregulated during aging, which was shown via heterochronic parabiosis studies (in which the blood circulation of young and old mice is connected). This connected blood system improves muscle, liver, brain and heart function^95^, ^96^ and reactivates stem cells in aged mice.^97^ Furthermore, heterochronic parabiosis reduces genomic instability in the aged mice^98^ and reverses expression profiles associated with aging.^99^ It is thought, that these rejuvenation effects are (partially) caused by some circulating factors in the blood of young mice. Several different factors, reduced in aged mice were identified, including GDF11,^96^ TIMP2,^100^ apelin,^101^ cadherin‐13,^102^ THBS4^103^ and SPARCL1.^103^ Infusion of exogenous GDF11 can restore heart,^96^ muscle^98^ and neural stem cell function in old mice,^104^ infusion of TIMP2 can increase cognitive function.^100^ The here proposed technology might be used to simultaneously produce several potentially rejuvenating factors or antibodies able to neutralize factors with potential pro‐aging effects, including CCL11,^105^ β2‐microglobulin^106^ and others.

For the abovementioned application, various therapeutic proteins must be simultaneously produced and adjusted to a defined concentration. Hence, the therapeutic protein concentration might be adjusted by therapeutic protein receptors able to recognize one of the produced therapeutic proteins. Adjusting the concentration of this one therapeutic protein would automatically control the concentration of the others. If the other therapeutic proteins need a different concentration the number of proteins produced by each cell might either be increased or decreased using differently active promoters.

## 7 CONCLUSION

The here proposed technology has to overcome several challenges (including its complex engineering process and potential immunogenicity) and the safety and longevity of the system must be proven. If these difficulties can be solved, the self‐regulating technology might enable a durable therapeutic protein expression at a specific concentration, making periodic protein infusions redundant. Thereby, the here proposed technology might be used as a platform technology to fight different diseases, including lysosomal storage diseases, haemophilia A/B and age‐associated diseases.

## AUTHOR CONTRIBUTIONS

**Philipp Schaible:** Conceptualization (lead); writing – original draft (lead); writing – review and editing (lead).

## CONFLICT OF INTEREST

The author confirms that there are no conflicts of interest.

## Data Availability

Data sharing not applicable to this article as no datasets were generated or analysed during the current study
